# Comparative efficacy and safety of fexuprazan versus esomeprazole in gastroesophageal reflux disease: a systematic review and meta-analysis

**DOI:** 10.3389/fmed.2026.1852781

**Published:** 2026-05-07

**Authors:** Omar Abureesh, Ali Sohail, Yousef Yousef, Abdullah Abureesh, Ahmad Abdulraheem, Ryan Tam, Ahmad Abou Yassine, Nadera Altork, Uday Sankar Akash Vankayala, Jean Chalhoub, Sherif Andrawes, Youssef El Douaihy

**Affiliations:** 1 Department of Internal Medicine, Staten Island University Hospital, Staten Island, NY, United States; 2 School of Medicine, The University of Jordan, Amman, Jordan; 3 Department of Internal Medicine, Medstar Washington Hospital Center, Washington, DC, United States; 4 Division of Gastroenterology and Hepatology, Department of Internal Medicine, University of New Mexico Hospitals, Albuquerque, NM, United States; 5 Division of Gastroenterology and Hepatology, Department of Internal Medicine, University of Nebraska Medical Center, Omaha, NE, United States; 6 Division of Gasstroenterology and Hepatology, Department of Internal Medicine, Staten Island University Hospital, Staten Island, NY, United States

**Keywords:** efficacy, esomeprazole, fexuprazan, GERD, meta-analysis, safety

## Abstract

**Background:**

Fexuprazan is a newly emerging potassium-competitive acid blocker (P-CAB) medication. P-CAB drugs have been introduced as alternative medications for gastroesophageal reflux disease (GERD) instead of traditionally used proton-pump inhibitors (PPIs).

**Aim:**

This study aims to compare the efficacy and safety of fexuprazan in contrast to esomeprazole.

**Methods:**

An electronic search was conducted on major databases, and retrieved results were subjected to two stages of filtration based upon a preset inclusion criterion. The first stage filtered articles based on their titles and abstracts, and the second stage filtered articles based on their full texts. Articles found fit to include in this study were extracted using a pre-set piloted extraction form. Extraction results were introduced into a spreadsheet. Data was divided into categorical and numerical data. Categorical data was reported as counts and frequencies, while continuous data was reported as proportions, means, and standard deviations. Common and random effect meta-analyses were conducted as found fit. Risk of bias assessment was performed using the Cochrane’s risk of bias assessment (RoB 2.0) tool.

**Results:**

Five articles were included in the present review, describing 965 patients managed with fexuprazan 40 mg or esomeprazole 40 mg for a mean of 9.6 weeks. The mean age of patients was 50.2 ± 13.0 years. GERD symptom scores showed a numerically lower value in the fexuprazan group (SMD − 0.41 [95% CI: −1.10; 0.29], *p* = 0.25), though this difference was not statistically significant and the confidence interval crosses the null (*I*^2^ = 94.5%, *p* < 0.01). The proportion of patients reporting symptom improvement did not differ significantly between groups (RR 0.85 [95% CI: 0.6; 1.22], *p* = 0.39; *I*^2^ = 91.3%, *p* < 0.01). No significant difference was observed in the incidence of treatment-emergent adverse events (TEAEs; RR 1.07 [95% CI: 0.9; 1.27], *p* = 0.45; *I*^2^ = 0.0%) or adverse drug reactions (ADRs; RR 1.10 [95% CI: 0.79; 1.53], *p* = 0.59; *I*^2^ = 0.0%).

**Conclusion:**

No statistically significant difference in efficacy was identified between fexuprazan and esomeprazole; however, preliminary findings are consistent with non-inferiority. No statistically significant difference in the incidence of TEAEs or ADRs was observed between treatments, suggesting a comparable safety profile.

**Systematic review registration:**

https://www.crd.york.ac.uk/PROSPERO/view/CRD420261286108.

## Introduction

Gastroesophageal reflux disease (GERD) is a common gastrointestinal problem affecting many around the world. In 2018, a study estimated that the global prevalence of GERD was around 13.3%, varying from 2.5% in China to 51.2% in Greece (1). This issue affects individuals causing abdominal upper middle quadrant pain, known as heartburn, in addition to regurgitation, dyspepsia, and bloating (2). The incidence of GERD is affected by many factors, including lifestyle, diet, weight gain or loss, pregnancy, and infections, especially *Helicobacter Pylori* bacteria, which cause gastritis (2).

Treatment of GERD includes dietary modifications, antacids or sucralfate, H₂-receptor antagonists, and proton pump inhibitors (PPIs) (3). The selection of therapy depends on several demographic and clinical factors; therefore, individualized patient evaluation is necessary to determine the severity of GERD and the most appropriate intervention. PPIs are widely used because of their availability and proven efficacy in GERD management. However, increasing concerns have emerged regarding potential safety issues associated with long-term PPI use. Reported adverse effects include fundic gland polyps, increased bone resorption, disruption of the gut microbiome, hepatotoxicity, and anemia (4). Esomeprazole was developed as a potent PPI and is commonly used in the management of GERD, including cases associated with *Helicobacter pylori* infection and other underlying pathological conditions (5).

Potassium-competitive acid blockers (P-CABs) have emerged as an alternative to PPIs, with the advantage of administration independent of meal timing and the potential to reduce complications commonly associated with long-term PPI therapy (6). Several systematic reviews and meta-analyses have examined the P-CAB class against PPIs in acid-related diseases broadly. Simadibrata et al. (2022) conducted a comprehensive meta-analysis across multiple gastric acid-related conditions, comparing all available P-CABs (vonoprazan, tegoprazan, keverprazan, and others) against PPIs, finding a comparable safety profile with a pooled risk ratio for treatment-emergent adverse events (TEAEs) of 1.13 [95% CI: 0.99–1.29] in favor of P-CABs (7). More recently, Simadibrata et al. (2024) specifically evaluated vonoprazan for PPI-resistant GERD, reporting mucosal healing rates exceeding 88% at 8 weeks (8). Zhuang et al. (2024) performed a network meta-analysis across 24 RCTs comparing P-CABs and PPIs for severe erosive esophagitis, finding vonoprazan ranked highest for both initial and maintenance healing (9). Collectively, these reviews establish compelling evidence base for P-CABs as a class; however, they encompass multiple agents and disease indications, and none has specifically examined fexuprazan, which is the most recently approved P-CAB in direct comparison with esomeprazole for GERD. The present review addresses this specific gap. Fexuprazan, a newly developed P-CAB, has demonstrated therapeutic potential in the treatment of erosive esophagitis and GERD, and has been approved or is currently undergoing regulatory approval in several countries (6). As a potential alternative to Esomeprazole, multiple studies have evaluated the efficacy and safety of Fexuprazan in comparison with established PPI therapy. This study aims to assess the available evidence comparing the efficacy and safety of esomeprazole and Fexuprazan in order to determine their relative effectiveness in symptom relief and their safety profiles with respect to adverse events and drug reactions.

## Methodology

### Study design

This systematic review was performed in accordance with the PRISMA checklist and Cochrane criteria (10, 11).

### Inclusion criteria

This study included clinical trials evaluating the efficacy and safety of Fexuprazan, a P-CAB, in comparison with the proton pump inhibitor Esomeprazole for the treatment of GERD. The primary outcomes of interest were improvement in GERD symptoms and the safety profile of the interventions. According to the predefined inclusion criteria, eligible studies met the following conditions: (1) Clinical trials that directly compared Fexuprazan with esomeprazole. (2) Completed Phase III blinded clinical trials. (3) Studies published in the English language.

### Search strategy and study selection

The PubMed/MEDLINE, Google Scholar, ScienceDirect including same database of Embase, and Scopus and CENTRAL databases were systematically searched from database inception to February 1, 2026. The search strategy included the terms “Fexuprazan” and “Gastroesophageal Reflux Disease (GERD).” For example, for ScienceDirect, the following query was used: “Title, abstract, keywords: fexuprazan AND (gastroesophageal reflux disease OR GERD OR erosive esophagitis) AND esomeprazole.” Detailed search strategies are provided in Supplementary File 1. Additionally, the full texts and reference lists of previously published systematic reviews on this topic were manually screened to identify potentially relevant studies. All identified records were exported to EndNote X9 reference management software after duplicate entries were removed. Study screening and data extraction were conducted independently by two reviewers, and any disagreements were resolved through discussion with a third reviewer.

### Risk of bias assessment

The methodological quality of the included studies was evaluated using the Risk Of Bias assessment tool 2.0 of Cochrane institute (12). This process was carried out by two sets of two reviewers, and discrepancies were solved by group discussion.

### Data acquisition

To minimize bias, data extraction was performed independently by two reviewers, and any disagreements were resolved by consultation with a third reviewer. The following data were extracted from each study: study design, country of participants, participant demographics (age and sex), description of the intervention, and primary outcomes related to GERD symptoms scores. Information regarding treatment-emergent adverse events (TEAEs) and adverse drug reactions (ADRs) was also collected. All procedures were conducted in accordance with the methodological recommendations outlined in the Cochrane Handbook for Systematic Reviews of Interventions (13).

### Data analysis

A planned meta-analysis using a random effects model was applied. Mean differences and 95% confidence intervals are shown. The collected data were analyzed by quantitative and qualitative methods. Patient and study characteristics were reported narratively in tables. Patient survival was analyzed using a fixed-effect model meta-analysis of proportion as the effect measure and 95% confidence intervals. Continuous variables were analyzed using standardized mean difference (SMD) or mean difference (MD) meta-analysis. Continuous GERD symptom outcomes were pooled using SMD, which assumes that the various symptom assessment instruments used across studies (e.g., RSI, GerdQ, total symptom score) measure the same underlying construct and that a one-standard-deviation change is clinically equivalent across scales. This assumption may not be fully held given the differing target populations and psychometric properties of these instruments, and the pooled SMD estimate should therefore be interpreted with caution. For binary outcomes, the risk ratio (RR) was selected as the primary effect measure. Statistical significance was set at *p* < 0.05. Heterogeneity was assessed using *I*^2^ and Cochran’s *Q* statistic, interpreted according to the Cochrane Handbook for Systematic Reviews. When significant heterogeneity was detected (*I*^2^ > 50%), we adopted a random-effects model. We identified studies causing heterogeneity using the leave-one-out method. If significant heterogeneity persisted after switching to a random-effects model, the relevant study was excluded from the synthesis. For the proportion of patients reporting symptom improvement, a pre-specified subgroup analysis was conducted based on the type of outcome measure used: (1) symptom-free day counts and (2) validated scale-based symptom scores. Statistical analyses were conducted using R software (version 4.12 of the meta package; R Foundation for Statistical Computing; Vienna, Austria). Assessment of publication bias using funnel plot asymmetry was not performed because the number of included studies in the meta-analysis of proportions was fewer than 10.

### Publication bias

Egger et al. claim that publication bias assessment is unreliable for less than 10 pooled studies. Because of this, we were unable to assess publication bias in the current study using Egger’s test for funnel plot asymmetry (14, 15).

### Protocol deviations

The present study was prospectively registered on PROSPERO (registration number: CRD420261286108). Several deviations from the registered protocol occurred and are transparently documented below. The registered protocol specified searches across six databases: PubMed/MEDLINE, Embase, CENTRAL (Cochrane Central Register of Controlled Trials), Scopus, Google Scholar, and ScienceDirect. The initial search was conducted across four of these databases (PubMed/MEDLINE, Google Scholar, ScienceDirect, and CENTRAL), as fexuprazan is a recently approved agent with a limited published trial base, and preliminary scoping confirmed that the majority of indexed trials were retrievable through these sources. Embase and Scopus were ultimately omitted to streamline the search after early literature saturation was observed. To ensure methodological rigor despite this omission, Google Scholar was leveraged, as it broadly indexes records from both of those databases. No additional eligible studies were identified through these supplementary searches, confirming the completeness of the original retrieval. The registered protocol listed quality of life and mucosal healing rate as secondary outcomes of interest. Neither outcome could be included in the quantitative synthesis for the following reasons: (a) quality of life was not assessed as a primary or secondary endpoint in the majority of included trials, and the single study reporting a quality-of-life measure used an instrument not replicated across other included studies, precluding pooling; (b) mucosal healing rate, where reported, was assessed using heterogeneous endoscopic grading systems (e.g., Los Angeles classification grades A–D, modified Hetzel–Dent scale), which could not be meaningfully combined across studies. These outcomes are described narratively where data permitted. The registered protocol stated that certainty of evidence assessment using the GRADE (Grading of Recommendations Assessment, Development and Evaluation) framework would not be performed. Upon peer review, we reconsidered this decision and have added a GRADE summary-of-findings table (Supplementary Table S2). Given the small number of included studies (*n* = 5), the high heterogeneity observed in efficacy outcomes, and the presence of one high-risk-of-bias study, the certainty of evidence was rated as very low for efficacy outcomes and low for safety outcomes. Readers are encouraged to interpret all pooled estimates in light of these certainty ratings.

## Results

### Search results

**Figure 1:**
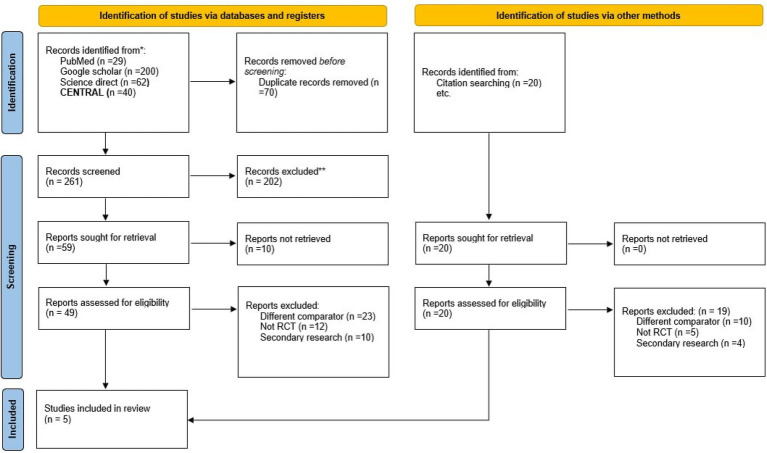
PRISMA chart of study filtration and inclusion.

The systematic—and other methods—search resulted in a total of 321 papers; following the removal of 30 duplicates, the remaining 281 studies were screened by title and abstract. Subsequently, 79 potential full texts were assessed for eligibility, resulting in a total of five papers that met the predefined criteria. The PRISMA flowchart illustrates the literature search, screening process, and reasons for exclusion (Figure 1).

### Baseline characteristics of the included studies

Included articles described 965 patients who were managed by Fexuprazan 40 mg or Esomeprazole 40 mg. On average, patients were managed for 9.6 weeks, and they were closely distributed among males and females (Males: 58.1%). The mean age of these patients was 50.2 ± 13.0 years. Tables 1, 2 summarize highlights of included articles and patients demographics. All studies except for one utilized a multi-centric RCT design, providing Fexuprazan 40 mg once daily as intervention.

**Table 1:** Characteristics of included studies.

Authors	Year	Duration (Weeks)	Sample size	Gender (M, %)	Age (mean ± STD)	Design
Lee et al. (21)	2022	8	263	152 (57.7%)	54.4 ± 12.7	Multi-center RCT
Kang et al. (23)	2025	8	161	58 (36%)	39 ± 12	Multi-center RCT
Oh et al. (24)	2025	16	39	16 (41%)	63.2 ± 8.2	Single-center randomized, open-label, prospective crossover trial
Kim et al. (22)	2024	8	170	73 (42.9%)	53.1 ± 13.4	Multi-center RCT
Zhuang et al. (25)	2024	8	332	262 (78.9%)	49.2 ± 12.3	Multi-center RCT
Total		9.6	965	561 (58.1%)	50.2 ± 13.0	

**Table 2:** Characteristics of included groups in this review.

ID	Year	Intervention	*N*	Age (Mean ± STD)	Timing of dosing
Lee et al. (21)	2022	Fexuprazan 40 mg	107	53.70 ± 12.44	Once daily, before a meal
		Esomeprazole 40 mg	111	55.05 ± 12.89	Once daily for 8 weeks
Kang et al. (23)	2025	Fexuprazan 40 mg	80	39.0 ± 12.0	Once daily, before a meal
		Esomeprazole 40 mg	81	40 ± 12	Once daily for 8 weeks
Oh et al. (24)	2025	Fexuprazan 40 mg	39	63.2 ± 8.2	Once daily, before a meal
		Esomeprazole 40 mg	19	62.8 ± 9.4	Morning, before breakfast
Kim et al. (22)	2024	Fexuprazan 40 mg	68	–	Once daily, before a meal
		Esomeprazole 40 mg	68	–	Once daily for 8 weeks
Zhuang et al. (25)	2024	Fexuprazan 40 mg	165	49.2 ± 12.3	Once daily, before a meal
		Esomeprazole 40 mg	163	46.9 ± 13.2	Once daily, before a meal

### Efficacy

**Figure 2:**
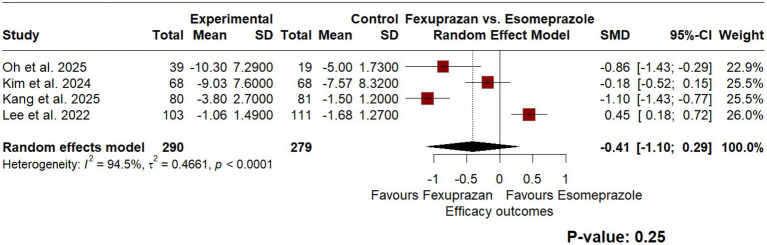
Forest plot of a random-effects model meta-analysis comparing the standardized mean difference (SMD) in GERD symptom scores between fexuprazan 40 mg and esomeprazole 40 mg. Significant heterogeneity was observed (*I*^2^ = 94.5%), indicating that these pooled efficacy estimates should be interpreted with caution.

**Figure 3:**
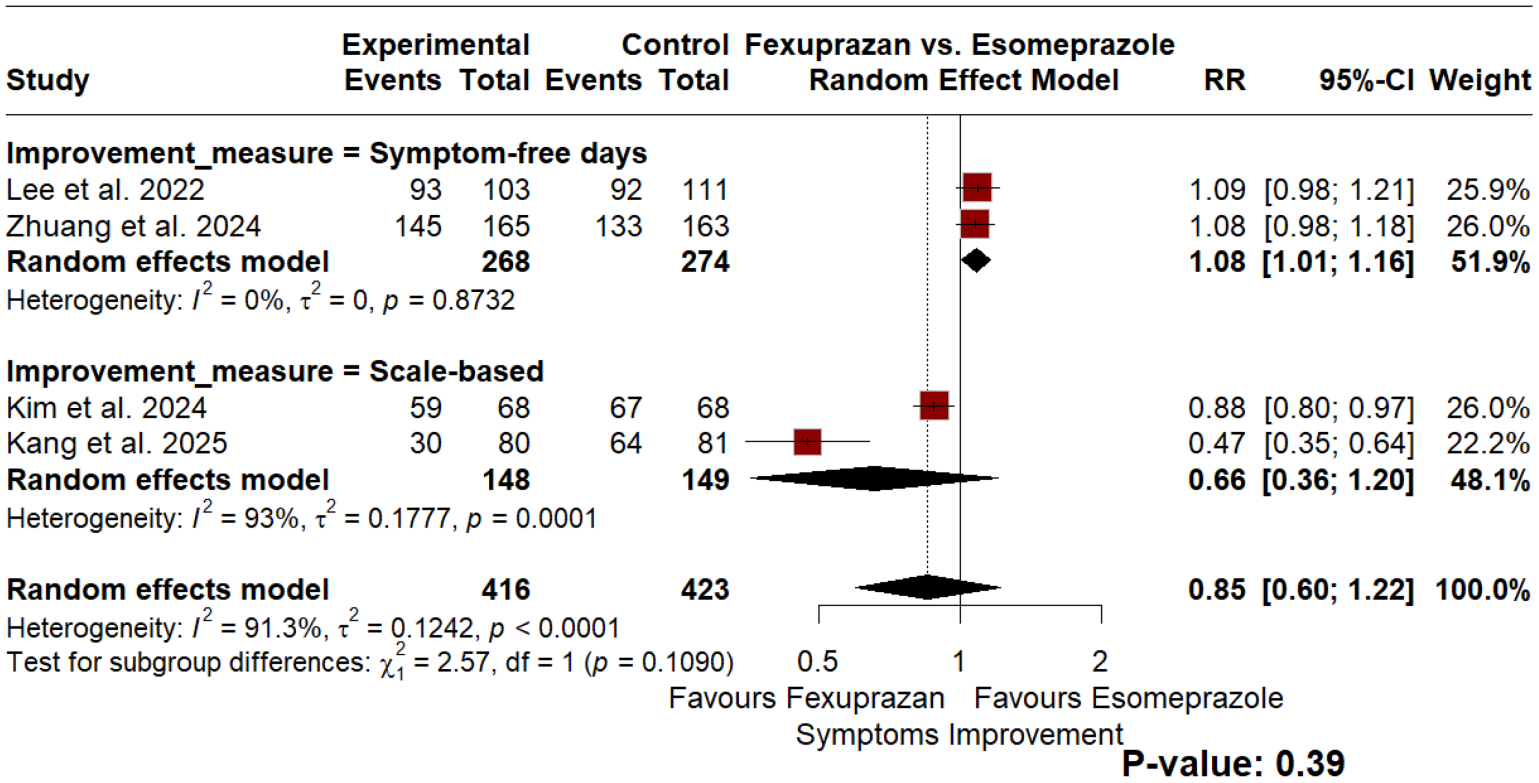
Forest plot of a random-effects model meta-analysis comparing the proportion of patients reporting symptom improvement between fexuprazan 40 mg and esomeprazole 40 mg, stratified by outcome measurement type. Studies are grouped into two pre-specified subgroups: (1) Symptom-free day counts (9, 19, 21) and (2) Scale-based symptom scores (22, 23). High heterogeneity persists (*I*^2^ = 91.3%), reflecting the clinical variability in outcome measurement tools across the included trials.

To compare the efficacy of each drug, a random effect model meta-analysis was conducted in articles with a quantitative assessment of GERD (Figure 2). Across studies, GERD symptom scores showed a numerically lower value in the fexuprazan group (SMD − 0.41 [95% CI: −1.10; 0.29], *p* = 0.25); however, this difference was not statistically significant and the 95% confidence interval crosses the null. Although standardized mean difference was used, heterogeneity remained significantly high (*I*^2^ = 94.5%, *p* < 0.01). A leave-one-out analysis excluding Oh et al. (24) was performed, since it was rated as high risk of bias. Exclusion of this study reduced efficacy heterogeneity to *I*^2^ = 78.3%, and the pooled SMD shifted to −0.28 [95% CI: −0.68; 0.13], remaining non-significant. Regarding symptoms improvement, the proportion of patients reporting symptom improvement did not differ significantly between groups (RR 0.85 [95% CI: 0.6; 1.22], *p* = 0.39), with the confidence interval crossing 1.0 in both directions. Similar to efficacy measures, results remained significantly heterogeneous (*I*^2^ = 91.3%, *p* < 0.01). Figures 2, 3 illustrate the forest plots for the meta-analysis of outcomes measures and symptom improvement, respectively.

### Safety

**Figure 4:**
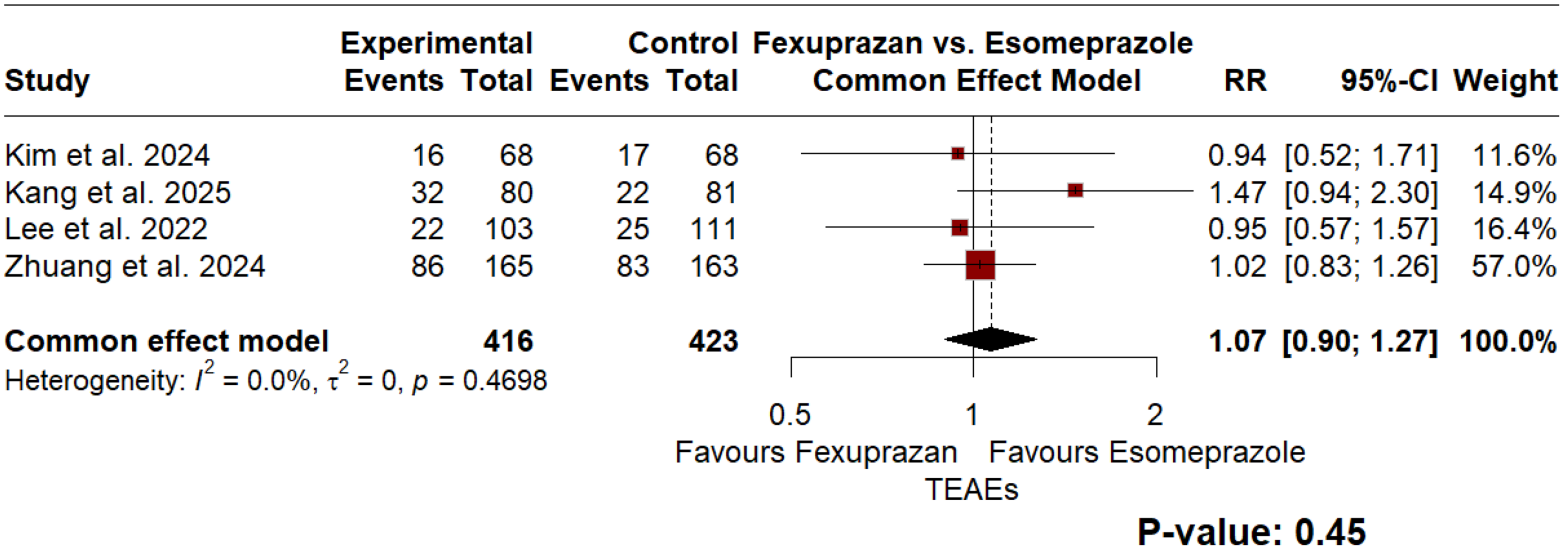
Forest plot of a common-effect model meta-analysis comparing the incidence of treatment-emergent adverse events (TEAEs) between fexuprazan 40 mg and esomeprazole 40 mg. The difference in the incidence of TEAEs between the two groups was not statistically significant (*p* = 0.45), indicating a comparable safety profile.

**Figure 5:**
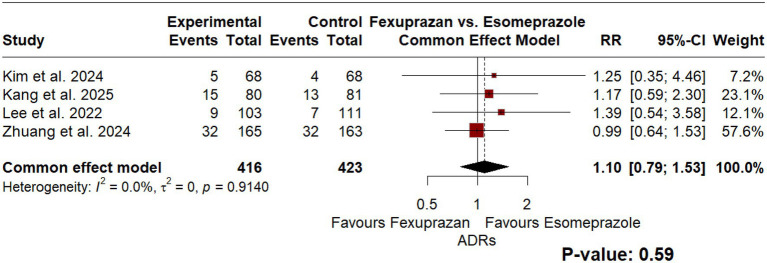
Forest plot of a common-effect model meta-analysis comparing the incidence of adverse drug reactions (ADRs) between fexuprazan 40 mg and esomeprazole 40 mg. The incidence of adverse drug reactions showed no statistically significant difference (*p* = 0.59) between treatments.

Regarding the safety of the intervention, comparing incidence of treatment-emergent adverse events (TEAEs) between the intervention and the comparator resulted in a RR of 1.07 [95% CI: 0.9; 1.27], *p* = 0.45, indicating no statistically significant difference in TEAE incidence between fexuprazan and esomeprazole. As for adverse drug reactions (ADRs), the comparison yielded a RR of 1.10 [95% CI: 0.79; 1.53], *p* = 0.59, indicating no statistically significant difference in ADR incidence between treatments, however; the model was insignificantly homogenous as well (*I*^2^ = 0.0%, *p* < 0.92). Figures 4, 5, represent the TEAEs and ADRs comparisons, respectively.

### Risk of bias assessment

**Figure 6:**
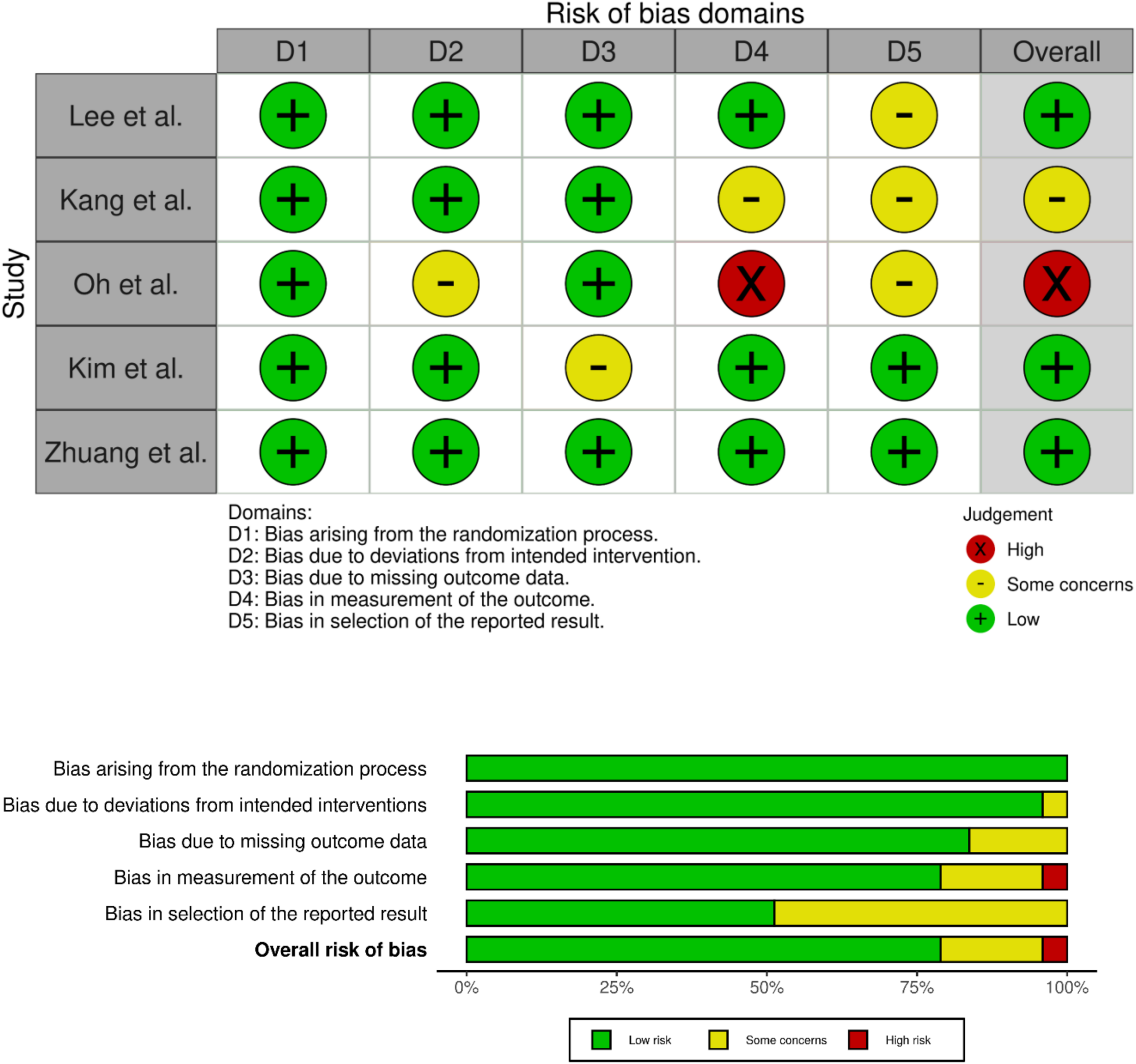
Risk of bias assessment of included studies.

Assessment of included papers for risk of bias results is presented in Figure 6. Overall, studies were of low risk of bias, except for Kang et al. which presented a moderate risk, mainly in the outcome measurement and selection of reported results domains, and Oh et al. (24) which was found to have high risk of bias due to lack of definitive measurement of outcomes.

## Discussion

Fexuprazan, a P-CAB medication developed recently to inhibit acid generation and secretion. The present study compared the efficacy and safety of this intervention to comparison to the traditionally utilized PPIs, specifically Esomeprazole. Pooled data in our study compared both interventions over an average of 9.6 weeks of 40 mg dosing one daily pre-meal. This modality falls within the recommended timeframe for short-term PPI treatment (16).

The efficacy of medical interventions in improving GERD or GERD symptoms is difficult to assess in a standardized manner. The diversity in diagnostic and assessment criteria makes the task of deterministically comparing different patient groups and interventions difficult. However, using a standardized mean difference (SMD) to analyze pooled estimates of change in different GERD assessment scores, the pooled estimate (−0.41 [95% CI: −1.1;0.29, *p* = 0.25]) does not demonstrate a statistically significant difference in efficacy between fexuprazan and esomeprazole; while the point estimate numerically favours fexuprazan, this should not be interpreted as evidence of superiority. Due to lack of standardized outcome measurement, meta-analysis of proportion was utilized to compare the proportion of patients reporting clinical improvement of GERD symptoms between groups. An RR of 0.85 [95% CI: 0.6; 1.22], *p* = 0.39 in favor of Fexuprazan was also reported, but was statistically insignificant, with high data heterogeneity (*I*^2^ = 91.3%, *p* < 0.01). The mechanism of action by which P-CABs, including Fexuprazan, cause their effect in acid reduction differs significantly from traditional PPIs. They are often described as potentially advantageous, due to their rapid, robust, and durable acid suppression, lack of CYP2C19 metabolism, independence from food intake, and non-requirement for activation (6). In addition, they have been suggested to restore small intestinal barrier integrity in mice, after NSAIDs-induced injury (17). Literature articles suggest that Fexuprazan is non-inferior to Esomeprazole, and our findings support this conclusion, although it remains in need of statistical and clinical confirmation with higher power analysis (6).

One major concern in traditional PPI therapy was their potential risk of developing medication-induced gastrin release (18). These effects have been avoided with the use of P-CABs, and literature suggests that a statistically significant lower release of gastrin has been recorded with use of P-CABs compared to PPIs (6). To add, comparing TEAEs proportions between Fexuprazan and Esomeprazole resulted in RR of 1.07 [95% CI: 0.9; 1.27], *p* = 0.45. Similarly, ADRs comparison estimated an RR of 1.10 for Esomeprazole over Fexuprazan [95% CI: 0.79; 1.53], *p* = 0.59, however; both models were insignificantly homogenous as well (*I*^2^ = 0.0%). Similar observations have been made by a meta-analysis by Simadibrata et al. (7). Their study compared the safety of P-CAB medications to PPIs in terms of safety profiles and found an overall OR of TEAEs of RR = 1.13 [95% CI.: 0.99; 1.29] favoring P-CABs to PPIs. These findings suggest that fexuprazan has a comparable safety profile to esomeprazole; no statistically significant difference in TEAEs or ADRs was identified, and the confidence intervals are consistent with either treatment being marginally safer. Some concerns have been raised toward the risk of hepatotoxicity when using P-CABs, however; available literature suggests that the drugs did not induce hepatotoxicity to higher extent compared to PPIs (6, 19).

Our findings are broadly consistent with those of prior systematic reviews examining P-CABs as a class. Simadibrata et al. (2022), whose analysis pooled multiple P-CABs across several acid-related conditions, reported a pooled risk ratio for TEAEs of 1.13 [95% CI: 0.99; 1.29] favoring P-CABs, a finding closely mirrored by our fexuprazan-specific estimate of RR 1.07 [95% CI: 0.91–1.26], neither of which reached statistical significance (7). This convergence supports the interpretation that fexuprazan’s safety profile is consistent with the broader P-CAB class profile rather than representing an outlier. With respect to efficacy, our results also align with the general class finding of non-inferiority to PPIs, as demonstrated across vonoprazan and tegoprazan trials. However, important distinctions between our review and prior class-level analyses merit emphasis. First, prior reviews include predominantly vonoprazan and tegoprazan data, agents with substantially larger trial bases and longer post-approval histories than fexuprazan. Second, our review is the first to restrict inclusion to Phase III RCTs specifically comparing fexuprazan against esomeprazole, providing a more focused and clinically direct comparison than class-level pooling allows. Third, the extreme heterogeneity we observed in efficacy outcomes (*I*^2^ = 94.5%) is largely absent in prior class reviews due to their broader scope and larger study pools, which underscores the need for standardized outcome measurement in future fexuprazan-specific trials.

Oh et al. (24) was the only study in this review rated as high risk of bias, primarily due to its open-label, crossover design and the absence of a definitive, pre-specified primary outcome measure, introducing the risk of carryover effects and period bias, performance and detection bias in the assessment of subjective symptom outcomes. Removal of this study reduced efficacy heterogeneity from *I*^2^ = 94.5% to *I*^2^ = 78.3%, and the pooled SMD shifted from −0.41 [95% CI: −1.10; 0.29] to −0.28 [95% CI: −0.68; 0.13], remaining non-significant (*p* = 0.21). Oh et al. (24) exerts a disproportionate influence on the pooled estimate, which is expected given that its mean difference and standard deviation were markedly larger than those of the other studies. Second, even after its exclusion, substantial heterogeneity persists, confirming that the instability of the pooled efficacy estimate is not attributable to this single study alone but reflects genuine clinical and methodological diversity across trials. Accordingly, the pooled efficacy estimate reported in this review should be interpreted as exploratory rather than definitive. Conclusions regarding the relative efficacy of fexuprazan versus esomeprazole cannot be reliably drawn from the current evidence base, and future trials should adopt standardized outcome instruments, parallel-group blinded designs, and adequate sample sizes to enable more robust synthesis.

A major limitation to the assessment of drug efficacy remains in the non-standardization of GERD diagnosis and assessment. The reliance on heterogenous outcome instruments resulted in significant data heterogeneity that could not be avoided. Concentrating on quantifiable GERD assessment criteria like the GerdQ score holds the utility to allow for more precise and powerful analysis (20). Another alternative is to rely on endoscopic assessment of GERD pre- and post-treatment, however; this method remains subject to individual interpretation and views. Another limitation is the lack of studies. As Fexuprazan is a relatively new medication, available literature investigating this matter is limited, and this compromises the ability to conduct important sensitivity analysis, publication bias, and meta-regression. Increasing the sample size for this comparison would allow for higher level detection of distinctions and factors. Additionally, the included patient populations were variable; encompassing erosive esophagitis, laryngopharyngeal reflux, and GERD-related cough. Furthermore, there was a notable lack of standardization in medication administration regimens across the trials (e.g., pre-meal versus morning dosing, as outlined in Table 2). This variability in dosing timing should be explicitly recognized as a significant clinical contributor to the extreme statistical heterogeneity (*I*^2^ > 90%) observed in the efficacy outcomes. Moreover, one of the trials included in this review had a high risk of bias (24), in addition to parallel RCTs, which should be considered when utilizing the results presented. Future research should focus on assessing the efficacy of P-CABs in comparison to PPIs in a standardized quantitative manner. Further, investigations with a deeper focus on the safety of P-CAB remain needed.

## Conclusion

Fexuprazan as a newly developed P-CAB medication, holds promising outcomes for GERD patients compared with traditional PPIs. Efficacy-wise, available data does not allow for confident conclusions, but points toward a non-inferior performance, pending further confirmation. The pooled efficacy estimate should be interpreted with caution given outcome heterogeneity, and that standardized use of validated tools such as the GerdQ in future trials would substantially improve the comparability of such analyses. Safety-wise, no statistically significant difference in the incidence of treatment-emergent adverse events or adverse drug reactions was observed between fexuprazan and esomeprazole. While point estimates slightly favored fexuprazan, these differences were not statistically significant and should not be interpreted as evidence of superior safety. The current body of evidence is insufficient to make confident efficacy claims, and the non-inferiority signal must be confirmed in future adequately powered and methodologically standardized trials.

## Data Availability

This is a meta-analysis based on published articles; extracted data from the original articles are available upon reasonable request from the corresponding author.
