# Supplementary material for: Comparative efficacy and safety of fexuprazan versus esomeprazole in gastroesophageal reflux disease: a systematic review and meta-analysis
Source: Front Med (Lausanne). 2026 May 7;13:1852781. doi: 10.3389/fmed.2026.1852781 (PMC13190402; doi:10.3389/fmed.2026.1852781)
Supplement: Supplementary file 1 [file Supplementary_File_1.docx]

**Search strategy:**

| Population:  P1 | “GERD” |
| --- | --- |
| P2 | “Erosive esophagitis” |
| P3 | “gastritis” |
| Intervention:  I1: | “Fexuprazan” |
| I2 | “Fexuclue”, |
| I3 | “Potassium-competitive acid blocker (P-CAB) “ |
| Comparator | “esomeprazole” |
| Outcome: O1 | “Nexium” |
| O2: | “Symptoms” |
| O3: | “recurrence” |

Search strategy:

Query 1: (P1 OR P2 OR P3) AND (I1 OR I2 OR I3) AND (C) AND (O1 OR O2 OR O3 )

Query 2: (P1 OR P2 OR P3) AND (I1 OR I2 OR I3)

Query 3: AND (I1 OR I2 OR I3) AND (O1 OR O2 OR O3 )
