# Supplementary material for: Comparative efficacy and safety of fexuprazan versus esomeprazole in gastroesophageal reflux disease: a systematic review and meta-analysis
Source: Front Med (Lausanne). 2026 May 7;13:1852781. doi: 10.3389/fmed.2026.1852781 (PMC13190402; doi:10.3389/fmed.2026.1852781)
Supplement: Supplementary file 2 [file Supplementary_File_2.docx]

**Supplementary Table S2**

| **GRADE Summary of Findings Table**  *Fexuprazan 40 mg vs. Esomeprazole 40 mg for Gastroesophageal Reflux Disease (GERD)* | | | | | | | | | |
| --- | --- | --- | --- | --- | --- | --- | --- | --- | --- |
| **Patient or population:** Adults with GERD (including erosive esophagitis, GERD-related chronic cough, laryngopharyngeal reflux, and nocturnal GERD symptoms)  **Setting:** Multicenter, single-center crossover trial  **Intervention:** Fexuprazan 40 mg once daily, before a meal  **Comparator:** Esomeprazole 40 mg once daily  **Included studies:** 5 clinical trials (Lee et al. 2022; Kim et al. 2024; Zhuang et al. 2024; Kang et al. 2025; Oh et al. 2025); n = 965 participants  **Follow-up:** Mean 9.6 weeks (range: 8–16 weeks) | | | | | | | | | |
| **Outcome** | **No. of Studies** | **No. of Participants** | **Events / Mean (Fexuprazan)** | **Events / Mean (Esomeprazole)** | **Effect Estimate (95% CI)** | **p-value** | **I²** | **Certainty of Evidence (GRADE)** | **Importance** |
| **EFFICACY OUTCOMES** | | | | | | | | | |
| **GERD Symptom Score Reduction (continuous; lower = better)** | 4 | 569 | Mean −5.54 (SD 5.45) | Mean −4.11 (SD 5.18) | SMD −0.41 [−1.10; 0.29] | 0.25 | **94.5%** | **⊕◯◯◯ Very Low (a)(b)(c)** | **Critical** |
| *Reasons for downgrading:* | (a) Serious risk of bias: 1 high-risk (Oh et al.), 1 moderate-risk (Kang et al.) study among 4 included (b) Serious inconsistency: I² = 94.5%, indicating extreme unexplained heterogeneity (c) Serious imprecision: 95% CI is insignificant; small total sample for pooled continuous analysis | | | | | | | | |
| **Proportion of Patients Reporting Symptom Improvement** | 4 | 839 | Symptom-free days: 238/268 (88.8%) Scale-based: 89/228 (39.0%) | Symptom-free days: 225/274 (82.1%) Scale-based: 131/230 (57.0%) | Overall RR 0.84 [0.56; 1.27] Symptom-free days: RR 1.08 [1.00; 1.17] Scale-based: RR 0.62 [0.48; 0.81] | Overall: 0.41 SFD: 0.06 Scale: <0.01 | **91.1% (0%; 0% by subgroup)** | **⊕◯◯◯ Very Low (a)(b)(c)(d)** | **Critical** |
| *Reasons for downgrading:* | (a) Serious risk of bias: same as above; (b) Serious inconsistency: extreme overall heterogeneity driven by qualitative difference between outcome types (symptom-free days vs. scale-based scores); (c) Serious imprecision: overall CI crosses null; (d) Indirectness: pooling of conceptually distinct improvement measures (symptom-free day counts vs. validated symptom scales) | | | | | | | | |
| **SAFETY OUTCOMES** | | | | | | | | | |
| **Treatment-Emergent Adverse Events (TEAEs)** | 4 | 839 | 156/416 (37.5%) | 147/423 (34.8%) | RR 1.07 [0.91; 1.26] | 0.41 | 0.0% | **⊕⊕◯◯ Low (e)(f)** | Important |
| *Reasons for downgrading:* | (e) Serious risk of bias: Oh et al. did not report TEAEs; only 4 of 5 studies contributed; (f) Serious imprecision: 95% CI crosses null (RR 1.07 [0.91; 1.26]); total events insufficient to exclude a clinically meaningful difference | | | | | | | | |
| **Adverse Drug Reactions (ADRs)** | 4 | 839 | 61/416 (14.7%) | 56/423 (13.2%) | RR 1.08 [0.77; 1.52] | 0.64 | 0.0% | **⊕⊕◯◯ Low (e)(f)** | Important |
| *Reasons for downgrading:* | (e) Serious risk of bias: same as above; (f) Serious imprecision: wide CI [0.77; 1.52] does not exclude a clinically relevant harm advantage in either direction; low absolute event rates limit the precision of estimates | | | | | | | | |
| **GRADE Certainty Key**  ⊕⊕⊕⊕ High — We are very confident the true effect lies close to the estimate.  ⊕⊕⊕◯ Moderate — We are moderately confident; the true effect is likely close but may be substantially different.  ⊕⊕◯◯ Low — Our confidence is limited; the true effect may be substantially different.  ⊕◯◯◯ Very Low — We have very little confidence in the estimate; the true effect is likely substantially different. | | | | | | | | | |

In accordance with the GRADE Working Group guidelines (Guyatt et al., BMJ 2011).
